# Multi-Material 3D Printed Shape Memory Polymer with Tunable Melting and Glass Transition Temperature Activated by Heat or Light

**DOI:** 10.3390/polym12030710

**Published:** 2020-03-23

**Authors:** Ela Sachyani Keneth, Rama Lieberman, Matthew Rednor, Giulia Scalet, Ferdinando Auricchio, Shlomo Magdassi

**Affiliations:** 1 Casali Center of Applied Chemistry, Institute of Chemistry and the Center for Nanoscience and Nanotechnology, The Hebrew University of Jerusalem, 91904 Jerusalem, Israel; 2 Department of Civil Engineering and Architecture, University of Pavia, 27100 Pavia, Italy

**Keywords:** shape memory polymers, 3D printing, 4D printing, multi-material printing, carbon nano tubes, actuators, soft robotics, finite element analysis

## Abstract

Shape memory polymers are attractive smart materials that have many practical applications and academic interest. Three-dimensional (3D) printable shape memory polymers are of great importance for the fabrication of soft robotic devices due to their ability to build complex 3D structures with desired shapes. We present a 3D printable shape memory polymer, with controlled melting and transition temperature, composed of methacrylated polycaprolactone monomers and N-Vinylcaprolactam reactive diluent. Tuning the ratio between the monomers and the diluents resulted in changes in melting and transition temperatures by 20, and 6 °C, respectively. The effect of the diluent addition on the shape memory behavior and mechanical properties was studied, showing above 85% recovery ratio, and above 90% fixity, when the concentration of the diluent was up to 40 wt %. Finally, we demonstrated multi-material printing of a 3D structure that can be activated locally, at two different temperatures, by two different stimuli; direct heating and light irradiation. The remote light activation was enabled by utilizing a coating of Carbon Nano Tubes (CNTs) as an absorbing material, onto sections of the printed objects.

## 1. Introduction

Among smart materials, shape memory polymers (SMP) attract great interest in the general field of materials science and, in particular, in soft robotics [1,2,3,4,5]. In combination with 3D printing techniques, SMPs can be used for fabrication of objects with complex geometry, which are capable of shape change and movement; this approach is also referred to as four-dimensional (4D) printing. SMPs, with activation triggered by heat, have been widely studied. The activation trigger can be direct heating [6,7,8,9,10], electric [11,12,13], magnetic fields [14,15] or light [16,17]. The advantage of magnetic and light activations is that they can be performed remotely, without the need of direct contact or wiring. For magnetic triggering, there is usually a need for a strong magnetic field induced by a robust electromagnet or magnet, while for light activation, a compact simple light source is sufficiently efficient. A variety of 3D printing methods have been demonstrated over the years for SMP materials, including extrusion-based methods, such as fused modeling deposition (FDM) [8,9,15,17], direct ink writing (DIW) [7,11,12] and stereolithography (SLA) approaches, including laser sintering [6], Polyjet technology [13,18] and digital light processing (DLP, also known as vat polymerization printing) [10,19,20,21]. An advantage of SLA is the possibility of printing with high resolution, which makes it a very favorable method for building various functional 3D objects.

Nowadays, new SMPs were developed and various modifications of these materials were reported. An important advantage is the ability to control the transition temperature of the material that is directly related to the control of the activation temperature. Tuning the transition temperature can be achieved in several ways: Mixing two different materials at various ratios [22], by controlling the degree of the SMP crosslinking [23,24,25] or; changing the type of crosslinking agent [19,25]. The first approach is suitable for Polyjet printing, in which the printer can print two different inks with controlled ratios at the same voxel. Other suitable approaches are based on vat polymerization printing, which has less limitations on ink properties and non-commercial inks can be easily applied.

In relation to the fabrication process, the possibility of printing a structure composed of various materials, by multi-material printing, is in principle, a great and unique advantage of fabrication by printing, also known as Additive Manufacturing (AM). Nowadays, while different approaches for multi-material printing are being developed, this technique remains a challenging process [26]. The common technology for multi-material printing is Polyjet printing [18,22,27], however, a major limitation of this approach is the requirement to utilize only low-viscosity commercial materials. Another unique way for multi-material printing is vat polymerization, in which either, the exposure time of the material can be controlled locally, as presented by Zhang et al. [23], or by replacing the vat during the printing process, as described by Ge et al. [25]. Although, the local exposure approach is highly interesting, only one layer can be exposed, which limits the ability to form 3D objects. The disadvantage of the vat exchange is the possibility of deposition of various materials only in the Z-axis direction and not in the X-Y plane. A novel and very promising approach was presented recently by Schwartz and Boydston [28], in which two different materials were printed in the same vat by two different polymerization mechanisms. This enables a structure, composed of two different materials, to be printed, without any spatial limitations.

The material selection and the fabrication method enable 3D models to be obtained, that can be triggered at two different temperatures. Thus, by a stepwise temperature-controlled movement, with specific control over the location of each material, unique locally programmable shapes can be formed. Ge et al. [25] first presented this approach by developing benzyl methacrylate-based SMP, with adjustable transition temperature, and demonstrated a multi-material gripper and a multi-material flower to be printed with two transition temperatures. The flower was activated by heating.

Another main challenge in SMP materials is obtaining a reversibility of the structural transitions. Typical SMPs can be activated one-way only, from temporary shape to initial shape. In order to re-shape the object to its temporary shape, an external force must be applied. Recently, efforts have been made to develop SMPs that can be activated in a two-way manner [29,30,31]. This can be achieved by photo-reversible crosslinks, integration of duel-actuation mechanisms or using semi-crystalline polymer networks. In the present paper, the reversible process of opening and closing a box is performed by developing 3D objects, composed of two materials having different transition temperatures.

Herein, we present multi-material 4D printing of SMP with tailored transition and melting temperature that can be activated both by direct heating and by light irradiation. The effect of the materials’ composition on the SMP behavior and mechanical properties was studied. A 3D printed double-lid box that opens at one temperature and closes at another temperature, simulating a controllable valve, is demonstrated. Supporting finite element analysis (FEA) predictions of the box movement are also presented. The box can be activated by direct heating or by light which enables remote activation.

## 2. Materials and Methods

**Ink formulation:** polycprolactone methacrylated (PCLMA) monomers were synthesized by alcohol–isocyanate reaction as described in our previous report [21]. N-Vinylcaprolactam (NVCL, Sigma-Alderich-Merck, Rehovot, Israel) was added to the PCLMA at different ratios. A photoinitiator, diphenyl(2,4,6-trimethylbenzoyl)phosphine oxide (TPO) of BASF, Ludwigshafen, Germany, in concentration 2.4 wt %) was added to the monomers. The mixture was placed in a heated sonication bath (60 °C) for 15 min, stirred and placed for additional 15 min in the heated sonication bath. This process was repeated until the photo-initiator was fully dissolved and the formulation was uniform.

**Thermal characterization:** The Differential Scanning Calorimetry (DSC, DSC 228, Mettler Toledo, Columbus, OH, USA) was used to measure the melting temperature of monomers with various NVCL:PCLMA ratios. The measurements were performed at various ranges between 0 to 120 °C, depending on the samples, at a heating rate of 1 °C/min. The samples were cooled before measurement. To form polymers with various NVCL:PCLMA ratios, the monomers were cured using UV light source (P1, Portable UV LED Curing system, 395 ± 5 nm, max. intensity 8 w/cm^2^). DSC measurements of the polymers were performed to detect the transition temperature. The measurements were performed at various ranges between 25 to 200 °C, depending on the samples, at a heating rate of 1 °C/min. The measurements were performed three times for each ratio (the error bars represent the standard deviation). Melting and transition temperature data were analyzed via one-way analysis of variance (ANOVA) to determine whether the different groups of an independent variable (i.e., the polymers with various NVCL: PCLMA ratios, in our case) have different effects on the transition temperature.

**Mold preparation for mechanical and SMP behavior tests:** Dog bone molds (dimensions according to ASTM D1708) of the polymers with different NCVL: PCLMA ratios were fabricated. The molding was carried out on a hot plate (60 °C), the sample was cured with UV light (P1, Portable UV LED Curing system, 395 ± 5 nm, max. intensity 8 w/cm^2^) for about 1 minute, released from the mold and additional illumination on the other side of the mold was carried out for 10–30 seconds.

**SMP behavior characterization:** The recovery and fixity values were calculated according to Lendlein et al. [32]. The samples were stretched to strain of ~40% using Universal Testing Machine (Instron 4502, Instron, Norwood, MA, USA), and the relevant lengths were measured. The measurements were repeated three times for each ratio (the error bars represent the standard deviation). Shape recovery and fixity data were analyzed via one-way ANOVA.

**Mechanical properties:** Mechanical properties were characterized by performing a tensile test below and above the transition temperature using a Universal Testing Machine (Instron 4502, Instron, USA) with 10 kN load cell, and a tension rate of 10 mm per minute. The Young’s modulus was calculated from the slope of the stress-strain curve (Figures S1–S12). The measurements were repeated three times for each ratio (the error bars represent the standard deviation). For 40 wt % NVCL above the transition temperature, 5 repetitions are presented. 

**Dual-material 3D printing:** Two inks were printed: PCLMA-only (will be addressed as pure ink) and 40 wt % NVCL:PCLMA composition (will be addressed as mixed ink). Both formulations were printed with a DLP printer (Pico2, Asiga, Alexandria, Australia) and were melted before printing. The pure ink was printed with a customized electrically heated bath, at a temperature of 95 °C. The mixed ink was printed while the printer was heated to 50 °C. The dual-material double-lid box was printed by pausing the printing and switching the baths. Six boxes were printed at once, each of the printing process was performed at least three times, and it was found repeatable. No post-treatment was required, only cleaning using a sonication bath heated to 60 °C when the samples are immersed in isopropanol. This cleaning process was repeated 4–5 times until the samples were clean without liquid residues.

**Heat triggered activation:** The SMP activation was done by immersing the 3D printed double-lid box in a bath of hot water, heated on a hot plate. The mixed polymer was triggered at 52 °C and the pure polymer was triggered at 58 °C.

**Light triggered activation:** For light activation, the double-lid box was coated with a CNT layer. Highly concentrated MWCNT ink was prepared by mixing 1.8 wt % of multi-walled carbon nanotubes, NC7000TM, (Nanocyl S.A., Sambreville, Bel-guim), a polymeric dispersant SOLSPERSE®46000 (3.6 wt %, Lubrizol, Wickliffe, OH, USA), and a wetting agent (0.1 wt %, Byk 348; Byk-Chemie GmbH, Wesel, Germany), in de-ionized water. The CNT in the ink was dispersed as describes elsewhere [33]. The coating of the inner and outer lid was done drop-wise: A drop of dispersion was placed on the lid followed by drying in an oven (60 °C). The inner lid was coated with ~12 drops and the outer lid with ~3 drops. The box was activated using UV light (P1, Portable UV LED Curing system, 395 ± 5 nm, max. intensity 8 w/cm^2^), and the temperature was recorded with a thermal camera (E4, FLIR, Wilsonville, OR, USA). The set-up of the measurement is presented in the supporting information (Figure S13).

**Modeling and simulation:** To predict the results of the heat triggered activation experiment on the 3D printed double-lid box, finite element simulations were performed by using the commercial finite element software ABAQUS (Simulia, Providence, RI, USA). The geometry was meshed by using eight-node linear isoparametric hexahedral elements with full integration. The thermo-mechanical response of the pure and mixed inks was captured by using the three-dimensional finite-strain constitutive model for SMPs, as proposed in [34]. Specifically, the model is based on a phase transition approach and formulated within a thermodynamically consistent mathematical framework. Moreover, the model is able to reproduce both shape-fixing and shape-recovery due to reversible state changes between glassy and rubbery phases, and it takes into account the non-ideal behavior of realistic SMPs (i.e., imperfect shape-fixing and incomplete shape-recovery). The model presents 11 material parameters that were calibrated on experimental data, as discussed in the next section. A quasi-static analysis was performed by imposing appropriate boundary conditions and a uniform temperature field history, as detailed in the next section.

## 3. Results and Discussion

**Figure 1:**
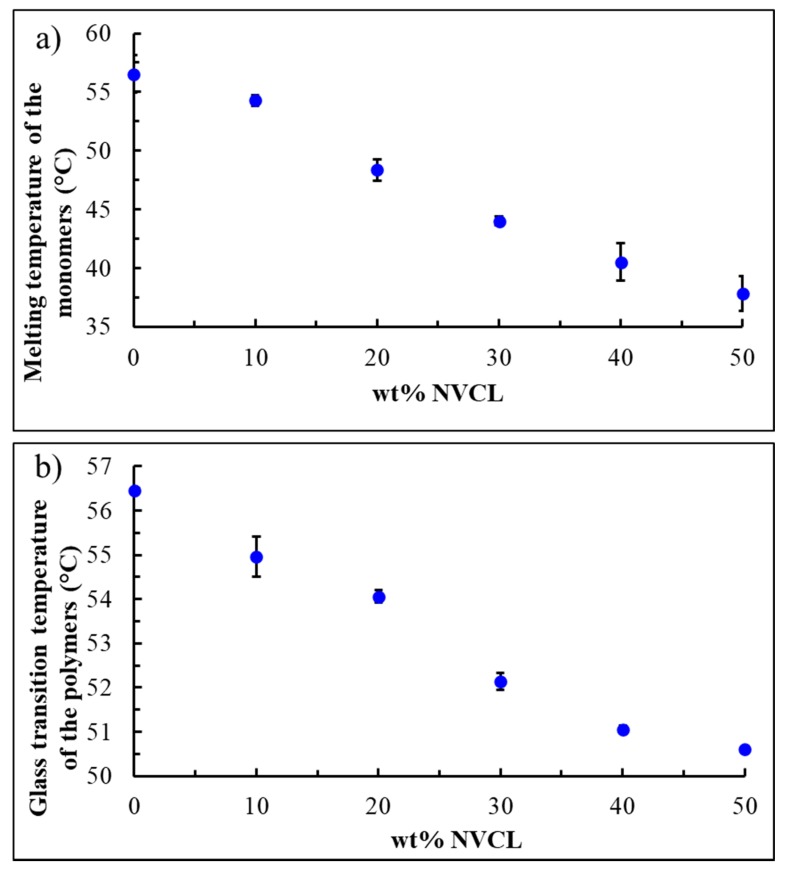
The effect of wt % of NVCL on the melting temperature of the PCLMA monomers (**a**) and on the transition temperature of the polymers (**b**). Three samples were tested for each experimental group.

*N*-vinylcaprolactam (NVCL) was added to the synthesized PCLMA as a reactive diluent (T_m_(NVCL) = 37.32 °C [35]), in order to control the melting temperature of our previously reported SMP monomers [21], and decrease it to the required temperature for mild condition printing. To characterize the compositions of PCLMA and NVCL with various weight ratios and to determine their melting temperatures, DSC measurements were performed (Figure 1a). The obtained results demonstrate that, as expected, the melting temperature decreases with the increase in NVCL content. For pure PCLMA, the melting temperature is 56 ± 2 °C while for a composition containing 50 wt % NVCL, the melting temperature decreases to 38 ± 1 °C, which is similar to the melting temperature of pure NVCL. Next, the effect of NVCL on the transition temperature of the polymer was studied. DSC data, presented in Figure 1b, clearly shows that the transition temperature of the polymer, as a function of NVCL content, has the same trend that was observed for the melting temperature. For pure PCLMA, the transition was observed at 56.46 ± 0.02 °C, while for composition containing 50 wt % NVCL it was found to be at 50.60 ± 0.02 °C. As followed from these results, NVCL has stronger effect on the melting temperature as compared with the transition temperature (the differences are ~20, and 6 °C, respectively). Nevertheless, as will be shown, such small but distinct difference in transition temperature enables the precise and high-resolution control over the activation temperature. To complete the discussion, the one-way ANOVA showed statistically significant differences in melting and glass transition temperatures, providing a *p*-value of 8.6912e-10 and 8.5564e-10, respectively. Accordingly, the small *p*-values are a strong indication that melting and transition temperatures differ significantly across polymers with various NVCL: PCLMA ratios.

**Figure 2:**
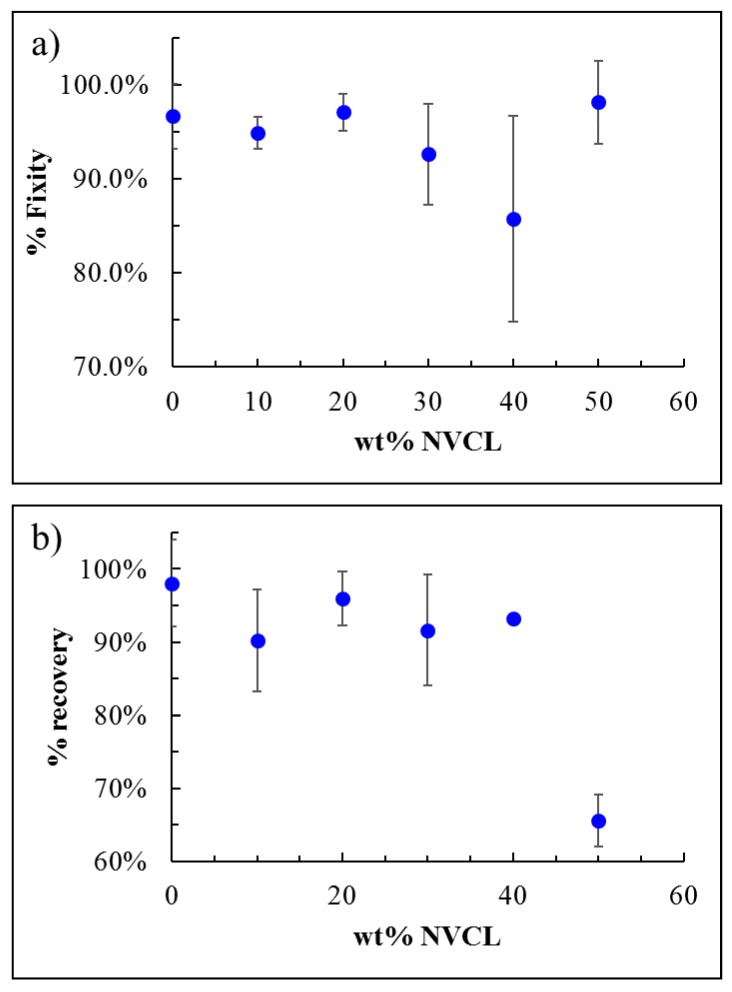
The effect of wt % of NVCL on the shape memory behavior: fixity (**a**) and recovery (**b**) Three samples were tested for each experimental group.

Once control over the transition temperature is achieved by changing the ink compositions, it is important to ensure that the behavior of the materials, such as SMPs, was not affected. Therefore, the recovery and fixity ratios were measured and calculated. As seen from Figure 2, the fixity remains good for all compositions and is in the range of 85%–100%, while the recovery ratio dramatically decreases at NVCL content above 40 wt %. Hence, at 1:1 NVCL: PCLMA weight ratio, the polymer acts purely as an SMP. Thus, in order to achieve maximal temperature separation with good SMP behavior, the pure PCLMA and the composition containing 40 wt % NVCL were chosen for the multi-material printing of the 4D objects.

To complete the discussion, the one- way ANOVA showed statistically significant differences in shape recovery performances (Figure 2b), providing a *p*-value of 9.7027e-05, and not statistically significant differences in shape fixity performances (Figure 2a), providing a *p*-value of 0.1468. 

**Figure 3:**
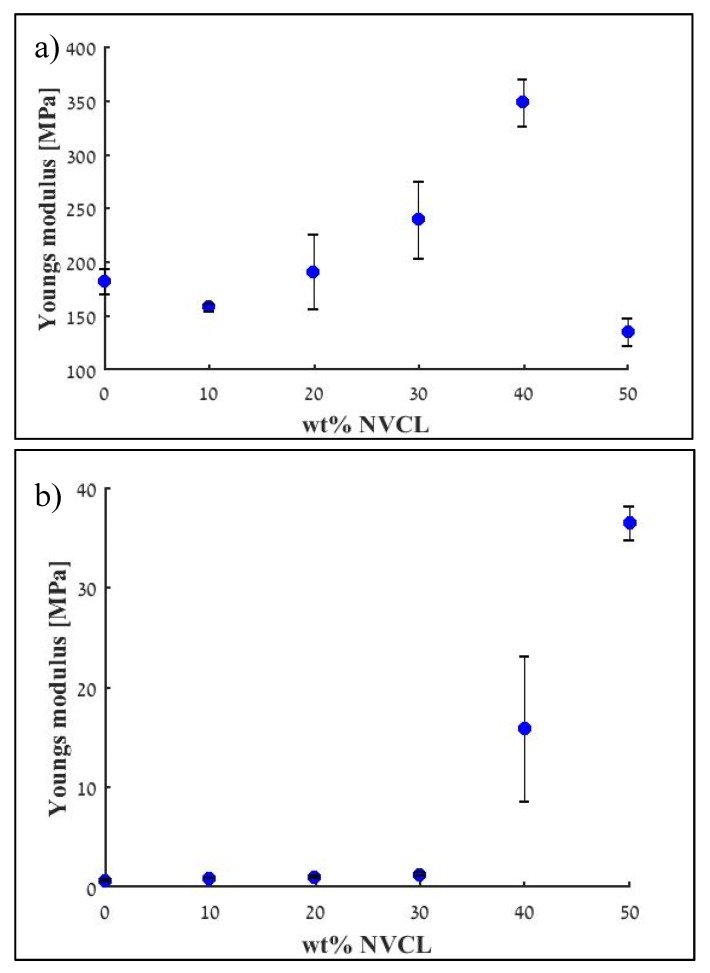
The effect of wt % of NVCL on the mechanical properties of the polymer: Young’s modulus for T < T_g_ (**a**) and for T > T_g_. (**b**). Three samples were tested for each experimental group, for 40 wt % NVCL above T_g_ five samples were tested.

The mechanical properties of the polymers, which are important characteristics of actuators and soft robotics, were also evaluated. The Young’s moduli at a temperature below (Figure 3a) and above (Figure 3b) the transition temperature for each polymer were calculated. As expected, Young’s modulus, below the transition temperature, was higher than the modulus above the transition temperature for all polymeric compositions. Below the transition temperature, the addition of NVCL up to 40 wt % NVCL results in a stiffer polymer, while further addition of NVCL causes a decrease in stiffness (as it can be seen for composition, containing 50 wt % NVCL). However, above the transition temperature, the addition of NVCL, up to composition containing 50 wt % NVCL, results in a stiffer polymer. This unexpected result is not fully understood and will be further investigated in a future study. 

It should be noted that the measurements of the Young’s modulus above the transition temperature for the compositions containing 40 and 50 wt % NCVL had large errors compared to the other compositions (Figure 3b). For samples containing 50 wt % NVCL, repeated measurements (performed for three different samples) resulted in reasonable errors (these are the measurements that are presented in the graph). However, for the 40 wt % NVCL, the errors remained large after the second repetition, so the Young’s modulus value was calculated by averaging the measurements of five samples (out of six, one sample was excluded). These are the values and error bars that are presented in Figure 3b. 

The calculated values of the transition temperatures and Young’s moduli for the pure PCLMA polymer and the sample containing 40 wt % NVCL were used for the calibration of the constitutive model employed in the numerical simulations.

**Figure 4:**
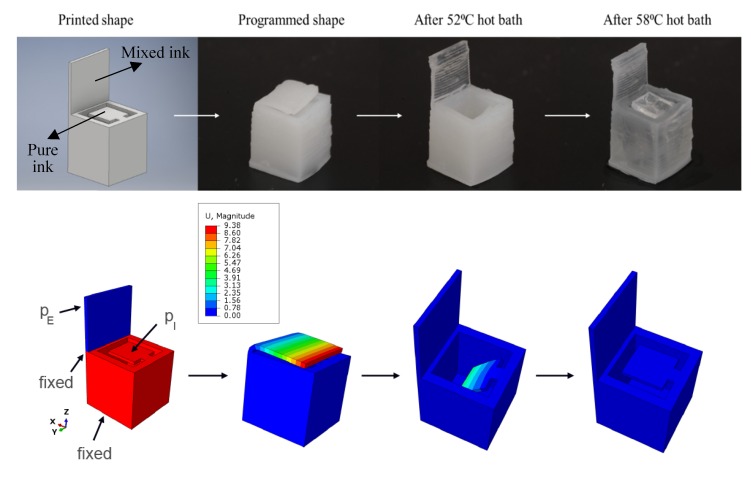
(**Top**) 3D printed box with two lids. The box opens at 52 °C and closes at 58 °C. (**Bottom**) Results of the finite element simulation. Red and blue colors in the first figure on the left denote pure and mixed ink, respectively. A pressure p_E_ of 0.0645 MPa and a pressure p_I_ of 0.00002 MPa were applied. Contour plots of the displacement magnitude [mm] are showed.

To demonstrate the dual activation process at two temperatures, a multi-material two-lid box was 3D printed (Figure 4). The bottom part of the box was printed with the PCLMA-only ink and the top lid was printed with the ink containing 40 wt % NVCL (“mixed ink”). The PCLMA-only ink was printed in a bath heated to 95 °C (at this temperature the ink is at its liquid form); while the mixed ink was printed at 50 °C. For multi-material printing, the baths were switched during printing, enabling printing objects composed of different materials in the Z-axis direction.

The thicknesses of the lids define the heat transfer ability through the material and thus the movement efficiency and recovery time. Therefore, after performing preliminary experiments, the selected thicknesses were 0.4 mm for the inner lid and 0.5 mm for the top lid.

The printed box was programmed at a temporary shape (by heating above 60 °C, re-shaping, holding and cooling at room temperature), in which the inner lid is open inwards, with an inclination level of 45°, and the top lid is closed. To test the two-temperature activation, the box was first activated by immersing in a hot water bath at 52 °C, that results in opening of the top lid only. Next, the box was immersed in a hot water bath at 58 °C, which causes the inner lid to close, thus achieving a full recovery of the box to its initial shape (Figure 4 and  Movie S1). It is interesting to note that when the material is heated above the transition temperature, it becomes transparent, which might have benefits in future optical applications.

A finite element analysis of the 3D printed box with two lids was performed, according to the description provided in the Modeling and Simulation section. Constitutive model parameters used in the analysis are listed in Tables S1 and S2 (in the supporting information) for the PCLMA, and the mixed inks, respectively. Particularly, Young’s moduli for the glassy and rubbery phases, respectively, E_g_ and E_r_, were defined from the data presented in Figure 3a, and 3b, respectively; Poisson’s coefficients for the glassy and rubbery phases, respectively, ν_g_ and ν_r_, were taken from ref. [34]; transformation temperature, θ_t_, was calibrated from values shown in Figure 1b; the parameter defining half-width of the temperature range, Δθ, and the transformation coefficient, w, were chosen taking into account that both inks are in a fully amorphous phase at 60 °C; the plastic hardening coefficient, h, and the stress limit, R^p^_g_, for plastic yielding of the glassy phase were calibrated from the stress-strain relationships below the transition temperature (see Figures S1 and S5) and above the transition temperature (Figures S7 and S11). No imperfect material behavior was assumed. Accordingly, coefficient c to tune imperfect shape-fixing was fixed equal to one, while coefficient c_p_ to tune incomplete shape-recovery was equal to zero.

The geometry, the coordinate system, material distribution, and the applied boundary conditions are provided in Figure 4 (bottom row). The bottom side of the box was fixed during all analyses. First, a pressure, p_I_, was imposed on the internal lid at 60 °C to open it inwards of an angle of 45°, while a pressure, p_E_, was imposed on the external lid at 60 °C to close it (the pressures were applied to simulate the shape programing step). In order to reproduce correctly the experimental deformation of the external lid, that did not affect the remaining part of the box, nodes at the interface of the two materials (see Figure 4) were fixed. Then, the deformed box was kept in position during cooling down to 25 °C. Finally, the deformed box was unloaded and re-heated up to 60 °C to induce shape-recovery. The results of the simulation are shown in Figure 4 (bottom). The external lid started opening at 51 °C and recovered the initial open configuration at 52 °C. The internal lid started moving at 56.5 °C and recovered the original closed configuration at 58 °C, in close agreement with the experimental findings.

**Figure 5:**
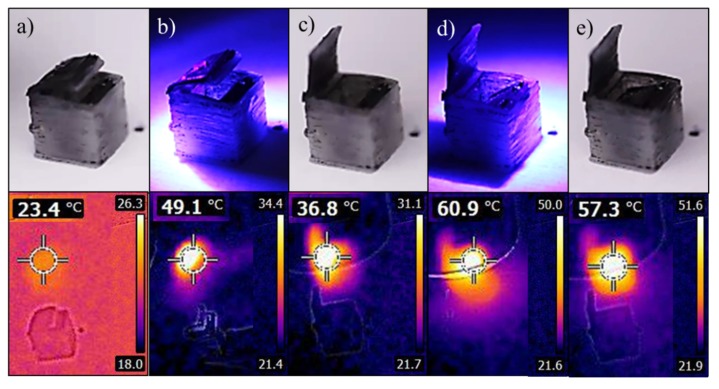
Images (**Top**) and thermal images (**Bottom**) of the two-lid-box with the light activation process. The programmed box (**a**) was activated by illumination with UV LED (395 ± 5 nm). First, the outer lid heated and opened, in about 1 minute (**b**,**c**) followed by the inner lid heated and closed, in about 8 min (**d**,**e**).

Finally, a 3D printed light-triggered multi-material SMP object was achieved by coating the lids of the box with a CNT layer, which absorbs light and transforms the light energy into heat. Thus, when the coated layer is exposed to light, it heats the objects, and therefore, local activation of the SMP is achieved. Since the top lid has lower transition temperature, it was coated with less layers of CNT. The light irradiation was performed at 395 nm LED source, since at this range the CNT absorb higher than at the visible light range [36]. The box was programmed as the same manner as before; where the inner lid is open inwards and the top lid is closed. Recovery is presented in Figure 5 and Movie S2, including thermal images. As expected, the activation of the top lid starts at around 49 °C, and the activation of the inner lid starts at about 60 °C. Light activation is favorable, compared to direct heat activation, since it does not require wet environment or any other direct heat source, it is a non-contact remote process and enables localized heating, thereby saving energy due to the localized activation.

It is important to note that the closing-opening process was performed over five times, so the process is reproducible.

## 4. Conclusions

Various compositions of 3D printable SMP inks with controlled melting and transition temperatures were developed. The difference in the transition temperature of the inks was several degrees, which enables a delicate control over the activation temperature. DLP multi-material 3D printing was performed, using two inks with different transition temperatures. With this approach, a printed box with two lids that open and close at two similar, but well distinguished temperatures, was fabricated. The box was triggered by two stimuli: Direct heating and light irradiation. Light activation is highly desirable, since it enables local, specific and remote activation. The research outcome has a potential for applications, such as in medical devices, as the box can simulate a valve or a pill for controlled drug release, or in soft robotics, by which remote actuation can be performed, while having different modes of movements at two different temperatures. However, work still needs to be done according to the specific application. For example, in drug delivery, the biocompatibility of the materials (especially the photoinitiators) and the in-body remote triggering should be further studied.

## Supplementary Materials

The following are available online at https://www.mdpi.com/2073-4360/12/3/710/s1, Figures S1–S12: detailed Young’s modulus analysis, Figure S13: Experimental setup for the cube’s light activation, Table S1: Constitutive model parameters adopted for modeling the pure ink (i.e., pure PCLMA) in the finite element analysis, Table S2: Constitutive model parameters adopted for modeling the mixed ink (i.e., ink composed of 40 wt% NVCL) in the finite element analysis, Video S1: A 3D printed two-lid-box with two transition temperatures activated by direct heating, Video S2: A 3D printed carbon nanotubes (CNTs) coated two-lid-box with two transition temperatures activated by light.
