# Supplementary material for: Multi-Material 3D Printed Shape Memory Polymer with Tunable Melting and Glass Transition Temperature Activated by Heat or Light
Source: Polymers (Basel). 2020 Mar 23;12(3):710. doi: 10.3390/polym12030710 (PMC7182824; doi:10.3390/polym12030710)
Supplement: Supplementary file 1 [file polymers-12-00710-s001.zip › Multi-material 3D printed shape memory polymer_supporting information_17.2.20_ES.docx]

**Multi-material 3D printed shape memory polymer with tunable melting and transition temperature activated by heat and light**

Ela Sachyani Keneth^1^, Rama Lieberman^1^**,** Matthew Rednor^1^, Giulia Scalet^2^, Ferdinando Auricchio^2^ and Shlomo Magdassi^1*^.

*1. Casali Center of Applied Chemistry, Institute of Chemistry and the Center for Nanoscience and Nanotechnology, The Hebrew University of Jerusalem, 91904 Jerusalem, Israel*

*2. Department of Civil Engineering and Architecture, University of Pavia, Pavia, Italy*

** Corresponding author.*

*Email addresses:* [*magdassi@mail.huji.ac.il*](mailto:magdassi@mail.huji.ac.il) *(S. Magdassi)*

**
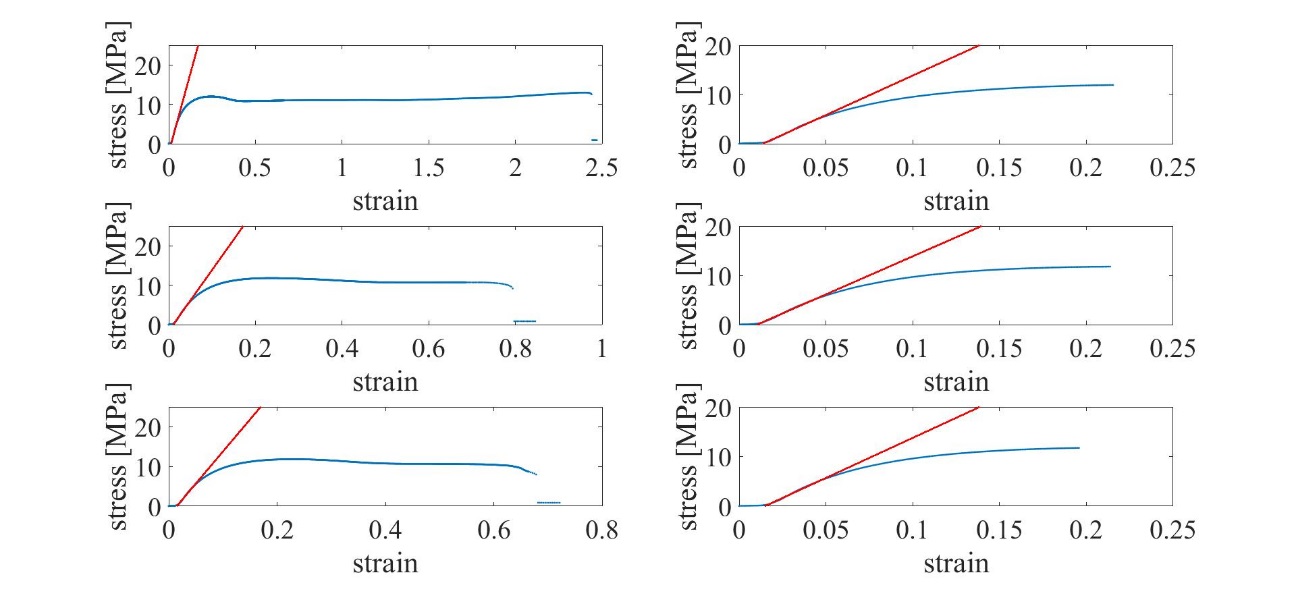

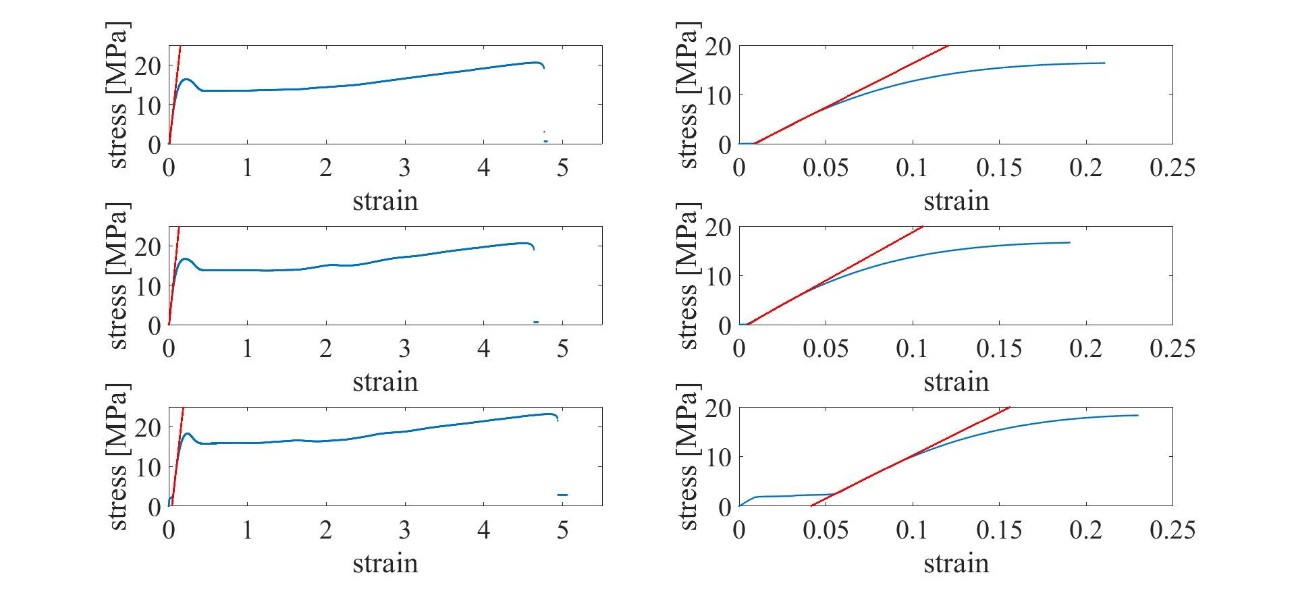
Supporting information**

***Figure S1:*** *Young's modulus analysis for pure PCLMA polymer below transition temperature.*

***Figure S2****: Young's modulus analysis for 10 wt% NCVL: PCLMA polymer composition below transition temperature.*

***
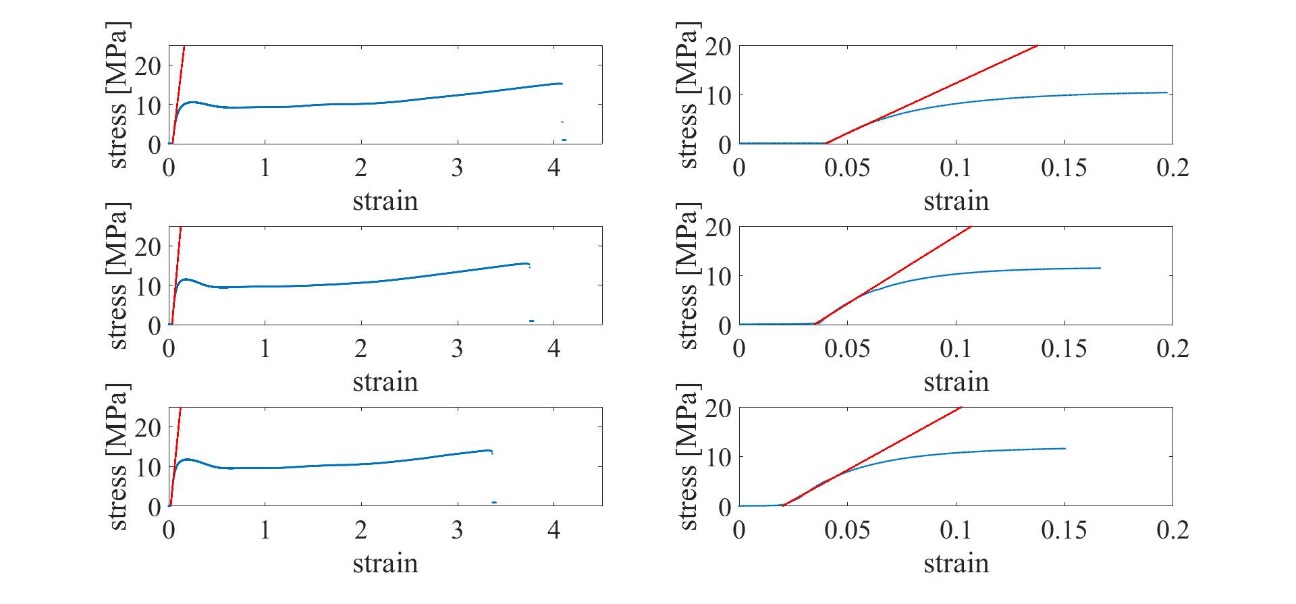
***
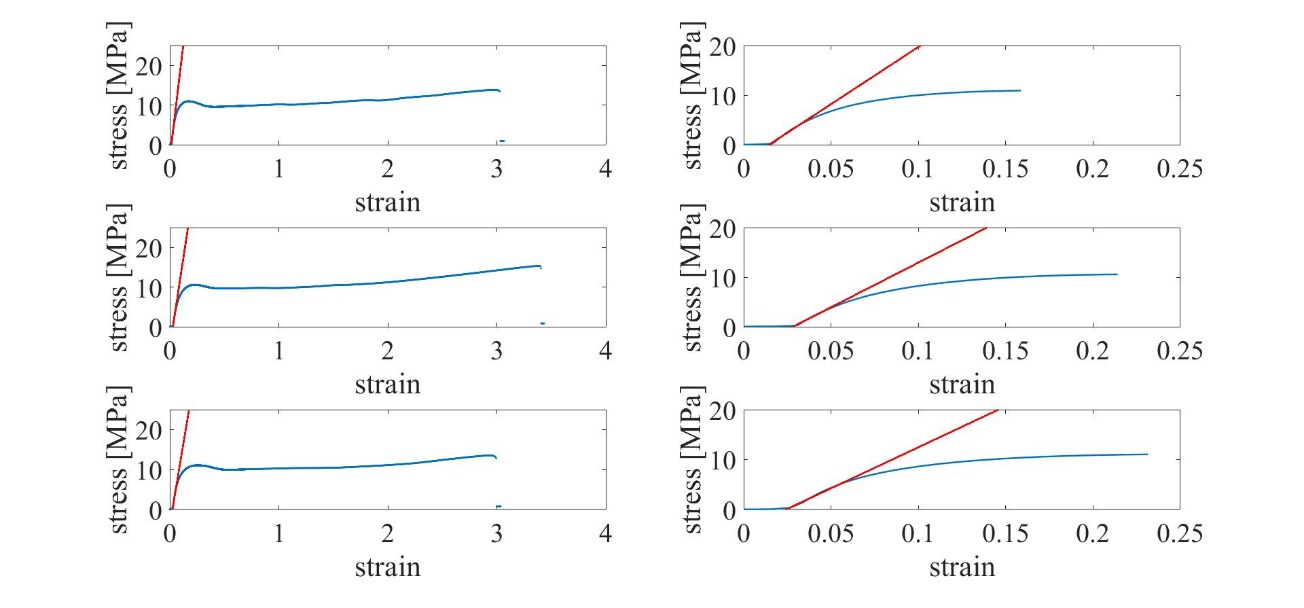
***Figure S3****: Young's modulus analysis for 20 wt% NCVL: PCLMA polymer composition below transition temperature.*

***
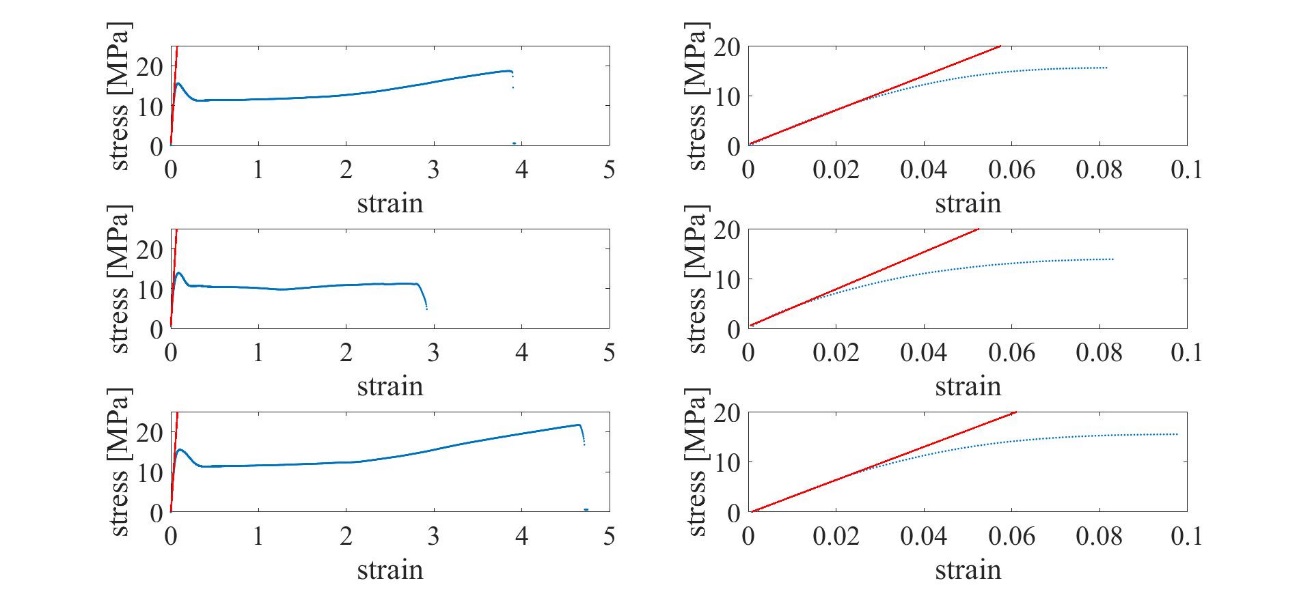
Figure S4****: Young's modulus analysis for 30 wt% NCVL: PCLMA polymer composition below transition temperature.*

***Figure S5****: Young's modulus analysis for 40 wt% NCVL: PCLMA polymer composition below transition temperature.*

***
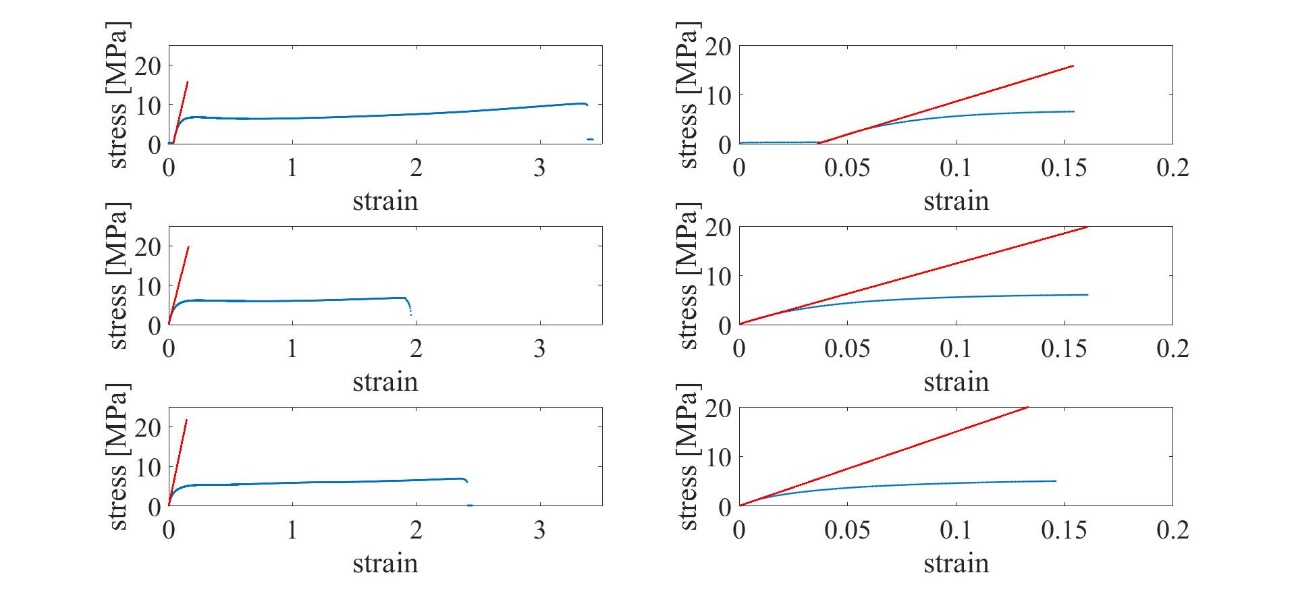
Figure S6****: Young's modulus analysis for 50 wt% NCVL: PCLMA polymer composition below transition temperature.*

***
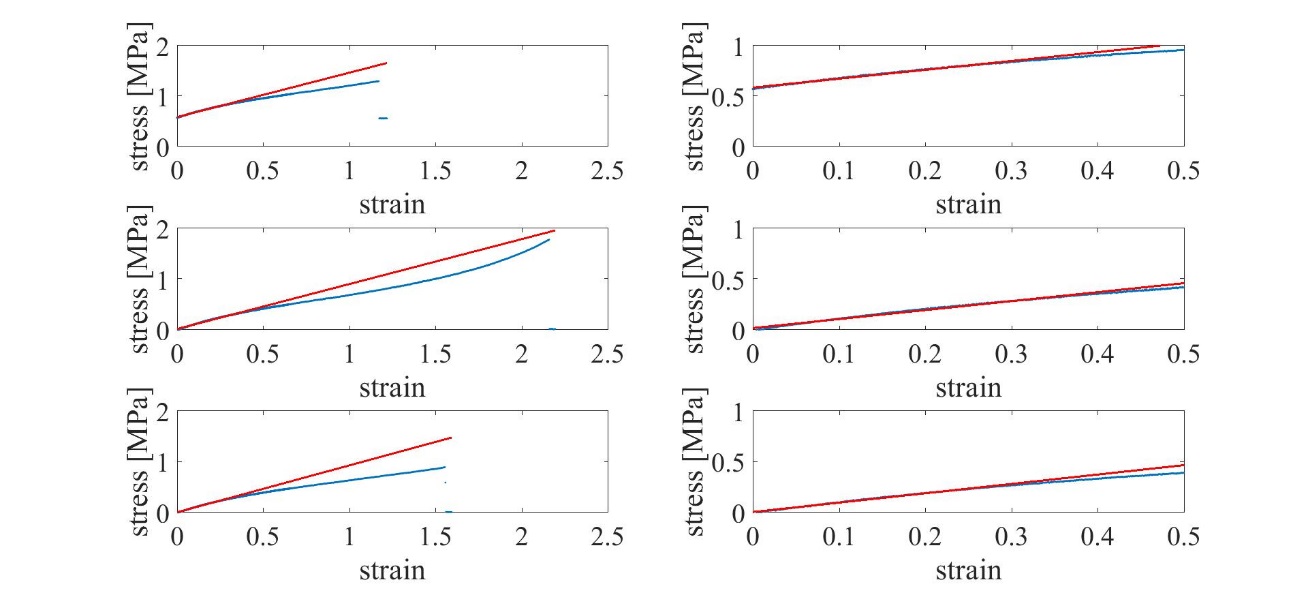
****
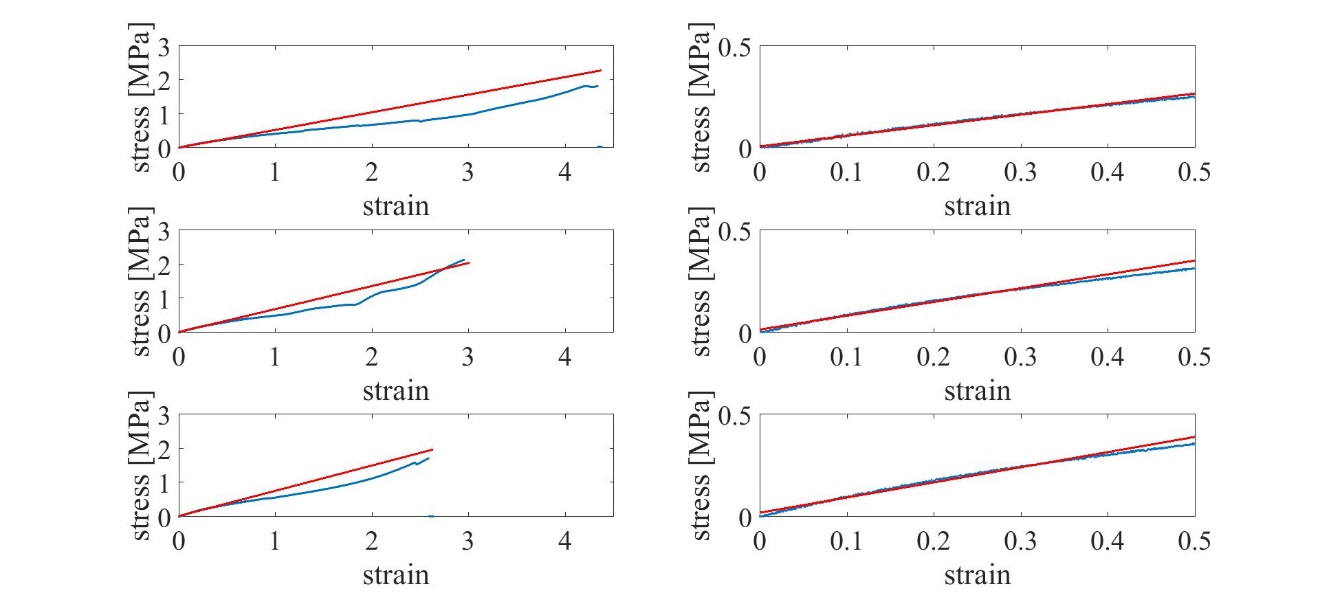
****Figure S7****: Young's modulus analysis for pure PCLMA polymer above transition temperature.*

***Figure S8****: Young's modulus analysis for 10 wt% NCVL: PCLMA polymer composition above transition temperature.*

***
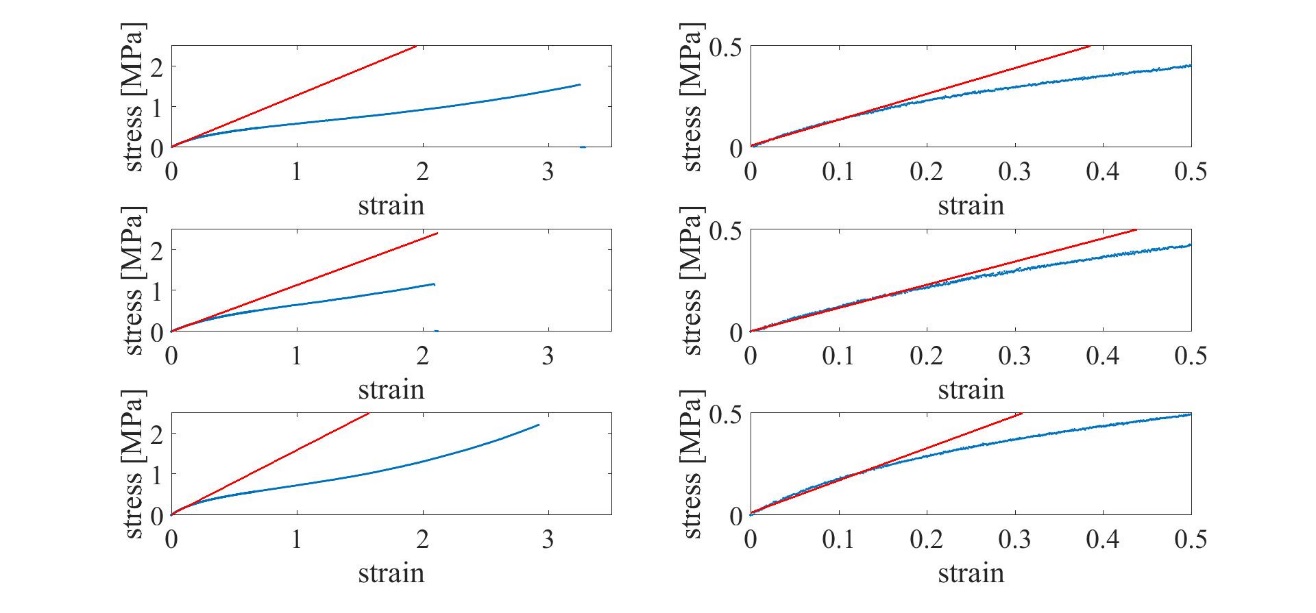
****
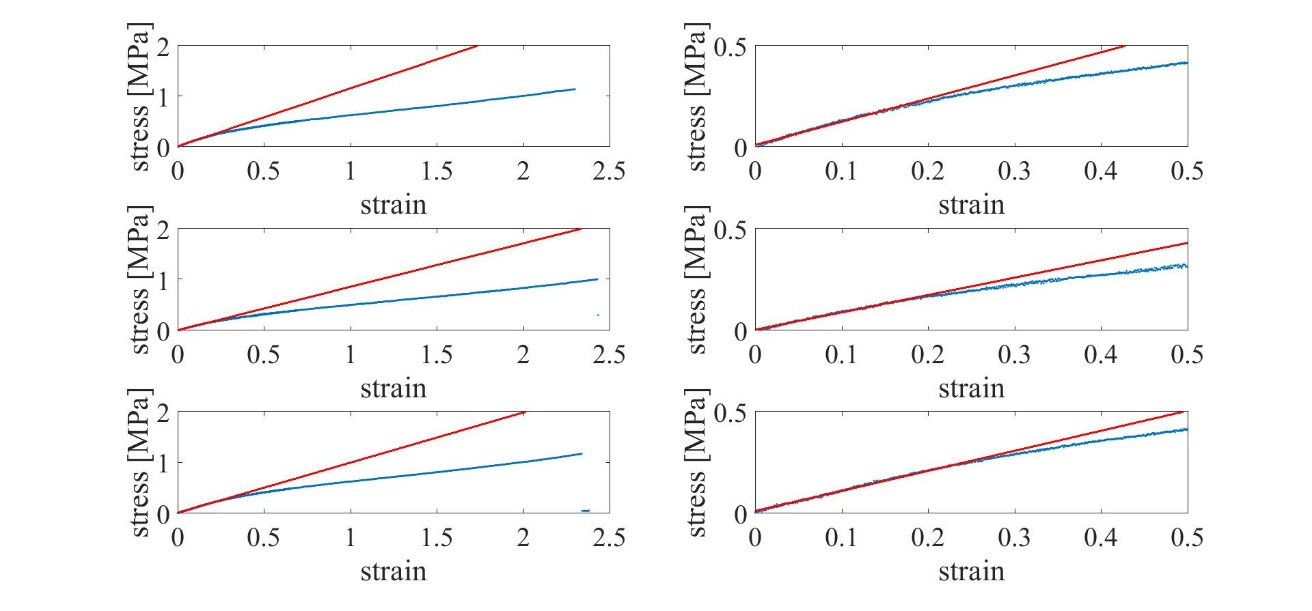
***Figure S9**: Young's modulus analysis for 20 wt% NCVL:PCLMA polymer composition above transition temperature.

**Figure S10**: Young's modulus analysis for 30 wt% NCVL:PCLMA polymer composition above transition temperature.

***
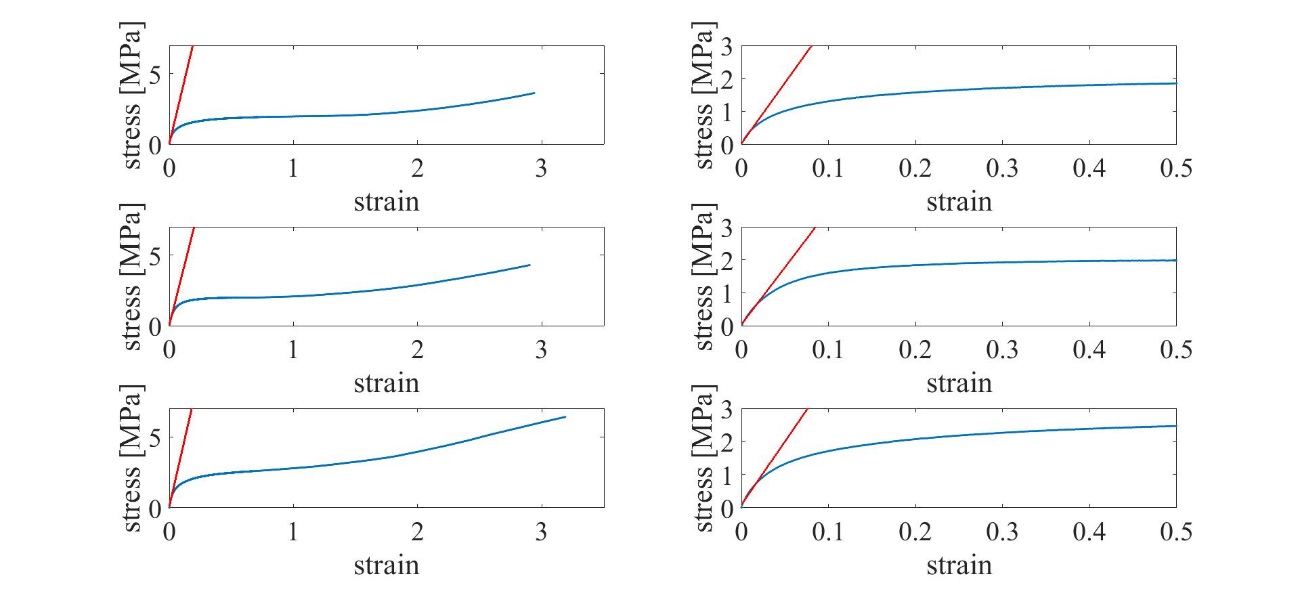
***


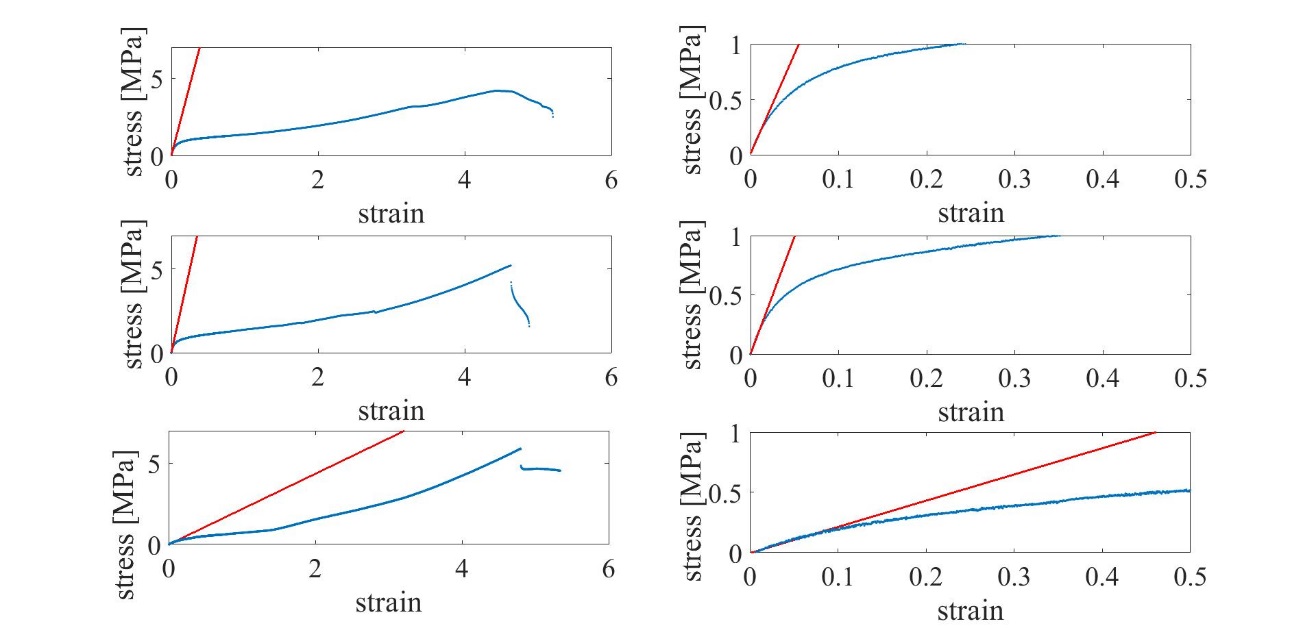

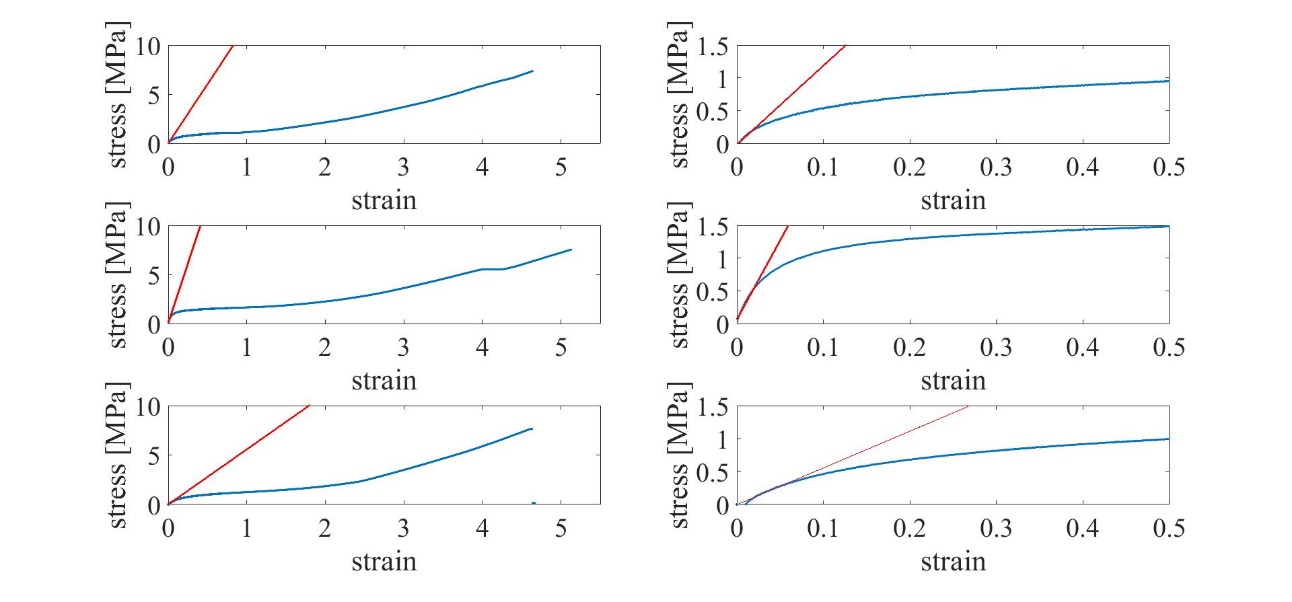


**Figure S11**: Young's modulus analysis for 40 wt% NCVL:PCLMA polymer composition above transition temperature

**Figure S12**: Young's modulus analysis for 50 wt% NCVL:PCLMA polymer composition above transition temperature

***
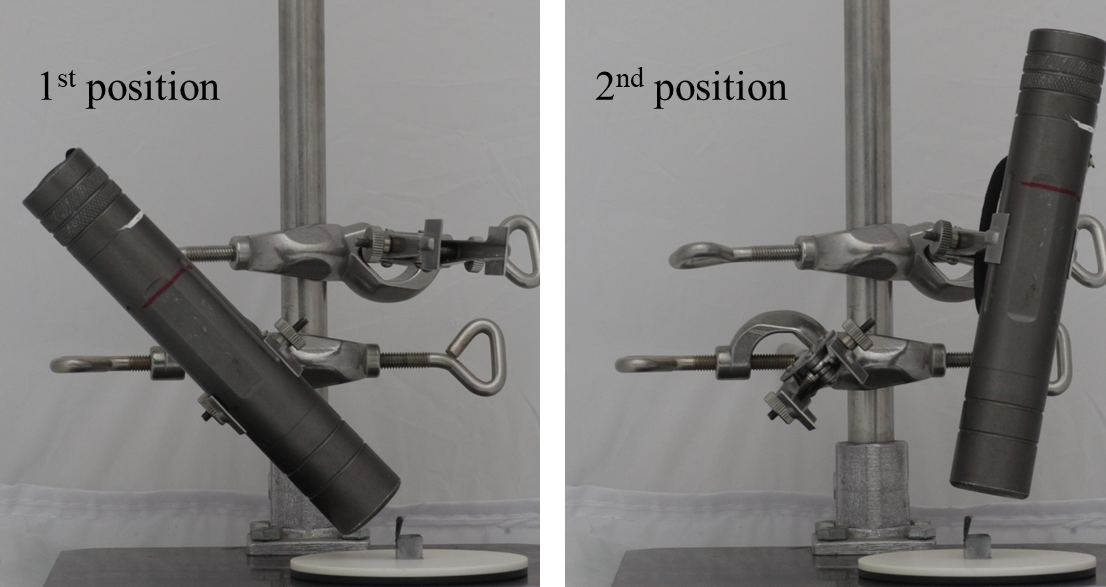
***

***Figure S13:*** *Experimental setup for the cube's light activation.*

***Table 1****: Constitutive model parameters adopted for modeling the pure ink (i.e., pure PCLMA) in the finite element analysis.*

| **Parameter** | **Symbol** | **Value** | **Unit** |
| --- | --- | --- | --- |
| Young’s modulus of the glassy phase | E_g_ | 185 | MPa |
| Young’s modulus of the rubbery phase | E_r_ | 0.25 | MPa |
| Poisson’s ratio of the glassy phase | ν_g_ | 0.29 | - |
| Poisson’s ratio of the rubbery phase | ν_r_ | 0.49 | - |
| Transformation temperature | θ_t_ | 56.5 | °C |
| Half-width of the temperature range | Δθ | 3 | °C |
| Transformation coefficient | w | 3.8 | 1/°C |
| Plastic hardening coefficient | h | 10 | MPa |
| Stress limit for plastic yielding of the glassy phase | R^p^_g_ | 15 | MPa |
| Imperfect shape-fixing coefficient | c | 1 | - |
| Incomplete shape-recovery coefficient | c_p_ | 0 | - |

***Table 2****: Constitutive model parameters adopted for modelling the mixed ink (i.e., ink composed of 40 wt% NVCL) in the finite element analysis.*

| **Parameter** | **Symbol** | **Value** | **Unit** |
| --- | --- | --- | --- |
| Young’s modulus of the glassy phase | E_g_ | 245 | MPa |
| Young’s modulus of the rubbery phase | E_r_ | 17 | MPa |
| Poisson’s ratio of the glassy phase | ν_g_ | 0.29 | - |
| Poisson’s ratio of the rubbery phase | ν_r_ | 0.49 | - |
| Transformation temperature | θ_t_ | 51 | °C |
| Half-width of the temperature range | Δθ | 3 | °C |
| Transformation coefficient | w | 3.8 | 1/°C |
| Plastic hardening coefficient | h | 10 | MPa |
| Stress limit for plastic yielding of the glassy phase | R^p^_g_ | 10 | MPa |
| Imperfect shape-fixing coefficient | c | 1 | - |
| Incomplete shape-recovery coefficient | c_p_ | 0 | - |

***Movie S1:*** *A 3D printed two-lid-box with two transition temperatures activated by direct heating*.

***Movie S2:*** *A 3D printed carbon nanotubes (CNTs) coated two-lid-box with two transition temperatures activated by light*.
